# The implementation of intrathecal morphine for caesarean delivery into clinical practice, and assessment of its impact on patient-reported quality of recovery using the ObsQoR-10-Dutch scale: A single-centre cohort study

**DOI:** 10.1097/EJA.0000000000002127

**Published:** 2025-01-29

**Authors:** Oscar F.C. van den Bosch, Mienke Rijsdijk, Suzanne E. Rosier, Lottie van Baal, Timme P. Schaap, Pervez Sultan, Wolfgang Bühre

**Affiliations:** From the Department of Anaesthesiology, Wilhelmina Children's Hospital (OFCvdB, SR, LvB, WB), Pain Clinic, Department of Anaesthesiology (MR), Department of Obstetrics, Wilhelmina Children's Hospital, University Medical Centre Utrecht, Utrecht, the Netherlands (TPS) and Department of Anaesthesiology, Perioperative and Pain Medicine, Stanford University School of Medicine, Stanford, California, USA (PS)

## Abstract

**BACKGROUND:**

Optimising a mother's quality of recovery following caesarean delivery is of paramount importance as it facilitates maternal care of the newborn and affects physical, psychological and emotional well being. Intrathecal morphine (ITM) reduces postoperative pain and may improve quality of recovery: however its widespread use is limited.

**OBJECTIVE:**

To assess the effects of implementing ITM for caesarean delivery on postoperative quality of recovery.

**STUDY DESIGN:**

Single-centre observational before–after study.

**SETTING:**

Tertiary university hospital, the Netherlands, January 2023 until April 2024.

**STUDY POPULATION:**

Patients who underwent caesarean delivery under spinal anaesthesia.

**INTERVENTION:**

Patients recruited before implementation of ITM (*n* = 55) received patient-controlled intravenous analgesia with morphine or continuation of epidural analgesia previously used for labour (’pre-ITM group’). Patients recruited after implementation of ITM (*n* = 47) received ITM 100 μg and oral morphine tablets 10 mg as needed (’ITM group’).

**OUTCOMES:**

Primary outcome was the score on the Obstetric Quality of Recovery (ObsQoR-10-Dutch) questionnaire (0 to 100). Secondary outcomes included ObsQoR-10 subscores, length of stay, opioid consumption and self-reported general health score (0 to 100).

**RESULTS:**

Protocol adherence for ITM was 98%. Quality of recovery improved significantly [ObsQoR-10 scores pre-ITM 65 ± 16 vs. ITM 74 ± 13 points, mean difference 9.0 (95% CI, 3.1 to 15] points, *P* = 0.002], with improvement in pain scores, physical comfort, independence and psychological wellbeing. In multivariate analysis, the improvement was 6.3 (95% CI, 0.37 to 12.2] points, which was statistically significant but did not reach the predefined threshold for clinical relevance. There was, however, an improvement in self-reported general health score (57 ± 18 vs. 68 ± 17, *P* = 0.002), median [IQR] length of hospital stay (41 [36 to 51] vs. 37 [32 to 49] h, *P* = 0.032) and median [IQR] opioid consumption (52 [35 to 73] vs. 0 [0 to 0] mg, *P* < 0.001).

**CONCLUSIONS:**

Implementing ITM for caesarean delivery resulted in moderate improvements in obstetric recovery and reduced opioid consumption. Cautious interpretation is warranted given the nonrandomised design of this implementation study. Our findings support the use of ITM in a multimodal analgesia strategy for patients undergoing caesarean delivery.

**KEY POINTS**

- Implementing intrathecal morphine (ITM) for caesarean delivery improves not only postoperative pain outcomes but also obstetric quality of recovery, through an improved ability to mobilise, to feed the newborn and feel more in control.
- Following implementation of ITM, there was a reduction in length of hospital stay and opioid consumption but an increase in pruritus.
- These findings were most pronounced following intrapartum caesarean deliveries and should be interpreted with caution given the observational nature of this implementation study.

## Introduction

Following caesarean delivery, we strive for excellent quality of recovery and optimal maternal wellbeing. Quality of recovery is considered a key outcome after caesarean delivery and is a multidimensional concept that includes physical comfort, physical independence, psychological support and emotional state.^1,2^ The postpartum period after caesarean delivery is complex and includes not only physical recovery from surgical stress but also the ability to take care of the neonate, taking care of a newborn, regaining physical independence and psychological wellbeing.

Inpatient quality of recovery after childbirth can be assessed reliably using the validated Obstetric Quality of Recovery (ObsQoR-10) score, rendering it a useful monitoring tool for interventions designed to enhance postoperative outcomes.^2,3^

The use of ITM for caesarean delivery is advocated by international guidelines based on evidence that it reduces postoperative pain and opioid consumption.^4,5^ However, previous research has primarily focused on unidimensional postoperative pain scores and opioid requirement rather than quality of recovery, which is a multidimensional construct.^6,7^ Even though effective analgesia may facilitate early mobilisation, breastfeeding and bonding, no studies address the effects of ITM on quality of recovery, beyond pain relief.^8^

Despite recommendations to use ITM from professional societies such as the European Society of Regional Anaesthesia and Pain Therapy,^4^ the Obstetric Anaesthetists’ Association,^4^ and the Society for Obstetric Anesthesia and Perinatology,^5^ concerns about side-effects such as nausea, pruritus and respiratory depression have limited the use of ITM across large parts of continental Europe and Asia. In a recent worldwide cohort study (’PAIN-OUT’), neuraxial long-acting opioids were administered in only 17.8% of caesarean deliveries.^9^ A better understanding regarding the effects on quality of recovery may therefore impact further adoption of ITM.

In this study, we aimed to translate and validate a Dutch ObsQoR-10 questionnaire for the population of the Netherlands and to assess the impact of implementing ITM for patients undergoing scheduled or intrapartum caesarean delivery under spinal anaesthesia.

## Methods

This was a single-centre observational cohort study before and after the institutional implementation of ITM for caesarean delivery as part of standard clinical practice. The study was conceptualised and commenced while reaching consensus within and between specialties in the hospital. The need for ethical approval by a Medical Ethics Committee was waived in accordance with Dutch law (Human Subjects Act). Instead, an independent quality check was carried out to ensure compliance with legislation and regulations regarding data management, informed consent and privacy aspects (ref. 23U-0149). All subjects were offered the option to object to the use of their data for research purposes. This study adheres to the STROBE statement on reporting observational studies.^10^

### Study design

This single-centre prospective cohort study was conducted at the Wilhelmina Children's Hospital, part of University Medical Centre Utrecht, the Netherlands. Patients aged 18 years or older who underwent scheduled and nonscheduled caesarean delivery under spinal anaesthesia were eligible to participate in this study. The exclusion criteria were conversion to general anaesthesia, midline incision and/or caesarean-hysterectomy, neonatal admission to NICU or intra-uterine foetal death, refusal to fill out the questionnaire, inability to read/understand Dutch and objection to the use of their data for research purposes. Patients were approached 24 [18 to 36] h after caesarean delivery by a resident or staff anaesthesiologist and asked to complete the ObsQoR-10-Dutch questionnaire (see Supplementary Material 1) and a global health visual analogue scale (GHVAS) score between 0 and 100 (0 = worst and 100 = best health state). We extracted from the electronic health records: patient characteristics (maternal age, weight, height, pre-operative haemoglobin level, chronic pain, ASA physical status, gestational age, gravidity and parity), caesarean delivery characteristics (scheduled or nonscheduled procedure, primary or repeat procedure, intrapartum procedure, postpartum haemorrhage) and postpartum recovery outcomes (length of stay from start of caesarean delivery until hospital discharge, nurse-recorded pain scores, oral morphine equivalents in 24 h, nausea and the use of antiemetics, pruritic and the use of antipruritics, and clinically significant respiratory depression). Patients before implementation of ITM were recruited into the ‘pre-ITM’ group from January 2023 until August 2023. The new protocol was commenced on 3 September 2023 and patients were recruited into the ‘ITM’ group from October 2023 until April 2024.

### Translation and validation of ObsQoR-10-Dutch

The ObsQoR-10 questionnaire was translated into Dutch and validated in accordance with previous validations in Turkish,^11^ Thai,^12^ French,^13^ Spanish,^14^ Hebrew,^15^ Portuguese,^16^ Hindi ^17^ and Arabic.^18^ Further details are given in Supplementary Material 2.

### Standard of care prior to intrathecal morphine

The pre-operative fasting interval at our institution is limited to 6 h for solids and patients are allowed to drink clear fluids up to the time of their caesarean delivery (up to 100 ml h^−1^). We routinely administer prophylactic vasopressors (phenylephrine 2 mg h^−1^) to prevent spinal anaesthesia-induced hypotension. The dose of hyperbaric bupivacaine as well as the use of off-label short-acting intrathecal adjuvants (fentanyl, sufentanil or clonidine) was left to the discretion of the attending anaesthesiologist. Normothermia was maintained with warm blankets and an operating room temperature of 23 °C. Two prophylactic intravenous antiemetics were given (ondansetron 4 mg and dexamethasone 8 mg) the latter also possesses analgesic properties. The first doses of paracetamol 1000 mg intravenously and diclofenac 75 mg intravenously were administered in the operating room after closure of the fascia. Postoperatively, standard of care included scheduled paracetamol 1000 mg p.o. every 6 h and diclofenac 50 mg p.o. every 8 h. In the postpartum ward, patients were offered ondansetron 4 mg intravenously when postoperative nausea and/or vomiting occurred. We routinely encourage patients to have early oral intake and promote early mobilisation after caesarean delivery.

Our postoperative analgesia regimen before the implementation of intrathecal morphine (’pre-ITM’) varied depending on whether or not an in situ epidural catheter had been used for labour analgesia. Patients without an in situ epidural catheter received patient-controlled intravenous morphine boluses of 1 mg (lockout 7 min, limit 12 mg per 4 h) for 24 h. For intrapartum caesarean deliveries with an epidural catheter in situ, our institutional practice was to administer single-shot spinal anaesthesia at least two interspace levels below the insertion site of the epidural catheter. The epidural catheter was left in place and epidural analgesia was continued postoperatively, using ropivacaine 0.1% + sufentanil 0.5 μg ml^−1^, 5 ml h^−1^, with additional patient-controlled epidural boluses of 5 ml, lock-out 10 min, limit 30 ml per 2 h.

### Implementation strategy

Several key steps were taken to optimise protocol design and adherence. Firstly, from January 2023 to March 2023, in-depth discussions were held in regular meetings of the anaesthesia department. Consensus was reached by presenting the underlying evidence and by making minor adjustments to the initial protocol to address practical concerns. Secondly, the pharmacy department was consulted, and vials of preservative-free morphine (100 μg ml^−1^, 1 ml) were stocked in the operating room to facilitate safe and easy administration of ITM. Thirdly, nursing leadership teams were involved to ensure the availability of rescue opioids, as well as antiemetics and antipruritics in the postoperative period. Other stakeholders, including obstetricians, neonatologists and the pre-operative clinic were involved and informed. Lastly, three educational meetings were held with obstetric nurses to explain the new protocol and answer any questions. The new protocol was officially launched in September 2023.

### Standard of care following implantation of intrathecal morphine

After implementation of the new protocol, ITM 100 μg (100 μg ml^−1^) was administered to patients during spinal anaesthesia for caesarean delivery. Postoperatively, in addition to scheduled paracetamol 1000 mg p.o. every 6 h and diclofenac 50 mg p.o. every 8 h, patients were offered morphine tablets 10 mg p.o. as rescue analgesia. The minimum interval between two morphine doses was 1 h and the maximum dose per 24 h was 60 mg p.o. The new protocol is in line with the procedure-specific postoperative pain management (PROSPECT) guideline for caesarean delivery endorsed by the European Society of Regional Anaesthesia and Pain Therapy and the Obstetric Anaesthetists’ Association.^4^

In the case of severe pruritus, patients were offered nalbuphine 5 mg i.v., which could be repeated after 1 h. Regarding monitoring of respiratory depression in the postpartum ward, those considered at low risk of respiratory depression were assessed clinically every 2 h for sedation and respiratory rate. Those considered at high risk of respiratory depression (BMI > 40 kg m^−2^, cardiopulmonary or neurologic comorbidity, known or suspected obstructive sleep apnoea) were additionally monitored with continuous pulse oximetry. Nalaxone was readily available in case clinically relevant respiratory depression occurred.

### Primary and secondary outcomes

The primary outcome of this study was the ObsQoR-10-Dutch score 24 h after caesarean delivery. The secondary outcomes were the sub-scores of the ObsQoR-10-Dutch questionnaire, GHVAS scores at 24 h, length of stay (from start of caesarean delivery until hospital discharge), mean nurse-recorded pain score over 24 h (NRS), need for (par)enteral opioids, opioid consumption from delivery to 24 h, nausea, need for additional antiemetics, pruritus, need for antipruritics and the incidence of clinically relevant respiratory depression.^19^ Protocol adherence in the ITM group was defined as the documented administration of ITM.

### Statistical analysis

A power analysis was performed to assess whether a sufficient number of participants would be recruited in the available time frame. A difference in ObsQoR-10-Dutch of 8/100 points was considered clinically relevant, in line with previous publications, which would require at least 41 participants per group, using a significance level of 0.05 and power of 80%.

Continuous data were summarised as mean ± SD or as median [IQR]). In univariate analysis, differences in ObsQoR-10-Dutch scores between the ‘pre-ITM’ and ‘ITM’ groups, as well as continuous secondary outcomes, were assessed with a student *t* test or a Mann–Whitney *U* test where appropriate. If a difference in the primary outcome reached statistical significance, the effect size was quantified with Cohen's *d*. The effect size is considered ‘*small’* when Cohen's *d* is 0.2 to 0.5, ‘*intermediate’* when Cohen's *d* is 0.5 to 0.8 and ‘*large’* when Cohen's *d* is more than 0.8. Categorical variables were compared between ‘pre-ITM’ and ‘ITM’ groups using χ^2^ tests. Linear regression analysis was used to assess the independent association between ITM and the ObsQoR-10-Dutch score, while controlling for potential confounders as previously defined in the literature (maternal age, body mass index, intrapartum and scheduled caesarean delivery). Opioid consumption was expressed as oral morphine milligram equivalents (MME; mg), using a conversion factor for intravenous morphine/oral morphine of 1 : 3.

Statistical analysis was undertaken with SPSS (Version 26, IBM) and R (R Core Team).

## Results

A total of 144 patients were approached and recruited for this study. After exclusions due to neonatal admission to NICU (*n* = 40), conversion to general anaesthesia (*n* = 1) and objection to the use of their data for research purposes (*n* = 1), the remaining 102 participants were included in the analysis. The cohort consisted of 55 patients pre-ITM and 47 patients in the ITM group. Protocol adherence in the ITM-group was 98%. Patient characteristics are presented in Table 1. Maternal age, weight or BMI did not differ between groups, whereas there were significantly fewer intrapartum and nonscheduled caesarean deliveries in the ITM group. In the pre-ITM group, 20% (*n* = 11) received postcaesarean epidural analgesia and their patient-reported 24 h pain score was similar to those without epidural analgesia (6.2 ± 2.0 vs 5.7 ± 3.1, *P* = 0.498).

**Table 1:** Characteristics of patients undergoing caesarean delivery under spinal anaesthesia before or after implementation of intrathecal morphine

	Pre-ITM (*n* = 55)	ITM (*n* = 47)	*P* value ^c^
Age (years)	32 [30 to 35]	33 [30 to 38]	0.117
Gestational age (weeks)	39.1 [38.0 to 40.1]	39.0 [38.2 to 39.3]	0.230
Weight (kg)	70 [62 to 82]	68 [59 to 78]	0.734
BMI (kg m^−2^)	26 [23 to 32]	25 [22 to 30]	0.528
Pre-eclampsia	3 (5)	7 (9)	0.180
Pre-operative haemoglobin (mmol l^−1^)	7.5 ± 0.8	7.5 ± 0.6	0.936
Chronic pain	1 (1.8)	0	
ASA physical status						0.843
2	40 (73)	35 (74)	
3	15 (27)	12 (26)	
Gravidity			0.593
1	19 (34)	12 (25)	
2	17 (31)	18 (38)	
3	13 (24)	8 (17)	
≥4	6 (11)	9 (19)	
Parity			0.367
0	22 (40)	14 (30)	
1	26 (47)	19 (40)	
2	5 (9.1)	9 (19)	
≥3	2 (3.6)	5 (11)	
Scheduled caesarean delivery	26 (47)	34 (72)	0.008
Intrapartum caesarean delivery	25 (45)	10 (21)	0.012
Repeat caesarean delivery	22 (40)	27 (57)	0.079
Postpartum haemorrhage	6 (11)	3 (6)	0.422
Spinal anaesthesia			
Hyperbaric bupivacaine dose (mg)	11.3 ± 1.1	11.8 ± 1.4	0.300
Sufentanil 2.5 μg	12 (22)	12 (25)	0.659
Fentanyl 20 μg	8 (15)	2 (4)	0.082
Clonidine 75 μg	17 (31)	3 (6)	0.002
None of the above	18 (33)	30 (64)	0.002
High neuraxial block, T3 or higher	0 (0)	0 (0)	N/A
Postoperative analgesia ^a^			
Morphine i.v. PCA	44 (80)	0	
Lumbar epidural analgesia ^b^	11 (20)	0	
Intrathecal morphine	0	46 (98)	

Data are shown as median [IQR], *n* (%) or mean ± SD. i.v., intravenous; ITM, cohort recruited after the implementation of intrathecal morphine; PCA, patient-controlled analgesia; pre-ITM, cohort recruited before the implementation of intrathecal morphine.

a In addition to scheduled paracetamol and diclofenac.

b Previously used for labour analgesia, continued postoperatively.

c Mann–Whitney *U* test, *t*-test or Pearson χ^2^ as appropriate.

### Validation of ObsQoR-10-Dutch: validity and reliability

The ObsQoR-10-Dutch questionnaire was validated similar to other translations, and a summary of the development and validation is provided in Supplementary Material 2.

### Effects of implementing intrathecal morphine on quality of recovery

Postpartum recovery outcomes are shown in Table 2. Quality of recovery as measured with ObsQoR-10-Dutch was significantly better in participants after implementation of ITM than in the pre-ITM group [74 ± 13 vs. 65 ± 16 points, mean difference 9.0 (95% CI, 3.1 to 15), *P* = 0.002, Cohen's *d* 0.617]. With regard to individual questions in the ObsQoR-10-Dutch questionnaire, scores improved significantly for pain, feeling comfortable, mobilising, holding the newborn, feeding the newborn, hygiene and feeling of control (Table 2). Postoperative opioid use was significantly lower following implementation of ITM; median [IQR, range] 52 [35 to 73, 17 to 171] vs. 0 [0 to 0, 0 to 44] mg, *P* < 0.001. Fewer patients required rescue analgesia in the ITM group (11 vs. 98%, *P* < 0.001). The incidence of nausea did not differ between the two groups (20 vs. 23%, *P* = 0.677). Pruritus occurred significantly more often following implementation of ITM (3.6 vs. 13%, *P* = 0.047). There were no cases of clinically relevant respiratory depression in either group.

**Table 2:** Postpartum recovery outcomes following caesarean delivery under spinal anaesthesia before and after implementation of intrathecal morphine

	Pre-ITM (*n *= 55)	ITM (*n* = 47)	*P* value ^d^
ObsQoR-10 at 24 h (0 to 100) ^a^	65 ± 16	74 ± 13	0.002
1 Pain	6 [4 to 8]	4 [2 to 7]	0.004
2 Nausea	1 [0 to 4]	0 [0 to 5]	0.247
3 Dizziness	1 [0 to 5]	2 [0 to 6]	0.434
4 Shivering	1 [0 to 5]	1 [0 to 4]	0.668
5 Comfort	7 [5 to 9]	8 [7 to 9]	0.039
6 Mobilisation	7 [5 to 8]	8 [6 to 10]	0.012
7 Hold baby	8 [6 to 10]	9 [8 to 10]	0.021
8 Feed baby	5 [3 to 8]	7 [5 to 8]	0.012
9 Hygiene	6 [3 to 8]	8 [5 to 9]	0.010
10 Control	6 [5 to 9]	8 [7 to 9]	0.007
GH-VAS at 24 h (0 to 100)	57 ± 18	68 ± 17	0.002
Length of stay (h) ^b^	41 [36 to 51]	37 [32 to 49]	0.032
Mean pain score over 24 h (NRS, 0 to 10)	2 [1 to 3.3]	1 [0.3 to 1.7]	<0.001
Oral morphine equivalents in 24 h (mg)	52 [35 to 73, 17 to 171]	0 [0 to 0, 0 to 44]	<0.001
Rescue analgesia	54 (98)	5 (11)	<0.001
Nausea	11 (20)	11 (23)	0.677
Additional antiemetics	4 (7.3)	9 (19)	0.073
Pruritus	2 (3.6)	6 (13)	0.047
Antipruritics ^c^	2 (3.6)	3 (6)	0.660
Respiratory depression	0 (0)	0 (0)	N/A

Data are shown as mean ± SD, median [IQR], median [IQR, range] or *n* (%). GH-VAS, general health measured by visual analogue score; ITM, cohort recruited after the implementation of intrathecal morphine; NRS, numeric rating scale; ObsQoR-10, Obstetric Quality of Recovery score; pre-ITM, cohort recruited before the implementation of intrathecal morphine.

a Sum of the 10 questions, scores of questions 1 to 4 are inverted.

b From start of caesarean delivery until maternal hospital discharge.

c Nalbuphine 5 mg i.v.

d Mann–Whitney *U* test, *t* test or Pearson χ^2^ as appropriate.

The effect of implementing ITM on quality of recovery was confirmed in multivariate analysis adjusting for potential confounders (maternal age, body mass index, scheduled caesarean delivery and intrapartum caesarean delivery) (Table 3). Implementing ITM was independently associated with a 6.3 (95% CI, 0.37 to 12.2) point increase in quality of recovery as measured by the ObsQoR-10-Dutch questionnaire. Intrapartum caesarean deliveries were associated with a 13.5 (95% CI, 0.93 to 26.1) point decrease in obstetric quality of recovery score (*P* = 0.036). Scheduled caesarean deliveries, maternal age and body mass index were not independently associated with different quality of recovery.

**Table 3:** Independent associations with quality of recovery (ObsQoR-10 score) 24 h after caesarean delivery in multivariate analysis

	Effect on ObsQoR-10 (0 to 100)	*P* value
Intrathecal morphine	+6.3 (0.37 to 12.2)	0.038
Maternal age	+0.45 (−0.19 to 1.1) per year	0.166
Body mass index	+0.15 (−0.35 to 0.64) per kg m^−2^	0.642
Intrapartum caesarean delivery	−13.5 (−26.1 to −0.93)	0.036
Scheduled caesarean delivery	−4.7 (−16.9 to 7.45)	0.443

Data (effects) are shown as beta coefficient (95% confidence interval). Independent effects on obstetric quality of recovery score in a linear regression model with ObsQoR-10-Dutch score as the dependent variable.

### Neonatal intensive care unit admission and maternal quality of recovery

In post hoc analysis, postpartum recovery parameters were compared between patients excluded because of neonatal intensive care unit (NICU) admission and those without NICU admission (Supplemental Material 3). Overall quality of recovery and general health scores did not differ significantly between patients whose baby required or did not require NICU admission. Quality of recovery subscores regarding the ability to hold or feed the baby were significantly better in patients without NICU admission.

## Discussion

In this study, we show a significant improvement in quality of recovery after caesarean delivery following institutional implementation of ITM. In multivariate analysis, although the extent of this improvement was 6.3 (95% CI, 0.37 to 12.2) points, it did not reach the predefined threshold for clinical relevance of 8 points. Other outcomes clinically relevant to the recovery period, such as self-reported health score, length of stay and the need for additional opioids in the postoperative period, were improved significantly.

The beneficial effects of ITM on postoperative pain have been studied extensively.^6^ In line with our results, a large body of evidence shows reduced pain scores, lower cumulative doses of additional opioids and improved patient satisfaction in patients receiving ITM.^20–27^ This has led to recommendations in peri-operative guidelines by the ‘PROSPECT’ Working Group of the European Society of Regional Anaesthesia and Pain Therapy (2021),^4^ the Society of Obstetric Anesthesia and Perinatology (2021)^5^ and the ERAS Society Guideline Committee (2019).^28^ These recommendations also acknowledge that the improved pain outcomes come at the expense of increased nausea and pruritus, which may hinder optimal recovery following caesarean delivery. A recent study showed the impact of implementing enhanced recovery measures on quality of recovery following caesarean delivery; however, ITM was already part of their clinical practice prior to practice change.^29^ To our knowledge, this is the first study to assess the impact of ITM on quality of recovery following caesarean delivery.

Concerns for side-effects hindering quality of recovery may be the reason that ITM is not consistently adopted worldwide. A retrospective analysis of a nationwide inpatient administrative-financial database in the United States showed 75.8% of parturients received ITM from 2008 to 2018.^30^ In contrast, analysis of a nationwide health insurance database in Japan showed that neuraxial morphine was used in only 20.8% of patients undergoing caesarean deliveries from 2005 to 2020.^31^ Nationwide surveys in Austria, Sweden and Israel revealed ITM utilisation rates of 5 to 63%.^32–34^ The international perioperative pain registry ‘PAIN-OUT’, undertaken in 105 centres worldwide showed that morphine was used in 17.8% of caesarean deliveries.^9^ A multicentre European snapshot study (’ACCESS’) is currently recruiting patients and will shed light on current anaesthesia practices for caesarean delivery across Europe.^35^

Our study shows that implementing ITM improved quality of recovery following caesarean delivery through improved pain, physical comfort, independence and psychological wellbeing, despite an increase in pruritus. The ideal dose of ITM in conjunction with other enhanced recovery measures remains a matter of debate and effective doses ranging from 50 to 250 μg were reported in a recent study.^36^ Side-effects of ITM are dose-related,^7^ and further work is needed to investigate whether a dose higher than 100 μg hinders the quality of recovery because of increased incidences of nausea and pruritis. Rates of clinically significant respiratory depression are reassuringly rare using contemporary dosing regimens.^19^

In addition to improvements in quality of recovery, the reduction in postoperative opioid consumption is striking. Even though rescue opioids must be offered to allow adequate recovery for those that need more analgesia, limiting the need for opioids is of great importance as this may prevent persistent postsurgical pain and persistent opioid use.^37–39^ Also, reducing the dependency on patient-controlled intravenous analgesia and epidural analgesia helps to allocate personnel more efficiently and to minimise the environmental impact associated with the production, disposal and energy consumption of the equipment and medications required.^40^

At our institution, spinal anaesthesia is routinely used for patients undergoing intrapartum caesarean deliveries with an in situ epidural catheter. Even though this practice is not common worldwide, it has been reported previously in the literature, with favourable outcomes.^41^ The advantages include optimal intra-operative conditions, less pain during caesarean delivery and fewer block failures compared with epidural anaesthesia. Reassuringly, there were no cases of high neuraxial block. The incidence of high neuraxial block is remarkably low in our region, where this anaesthetic practice is commonly used.^42^

The main strength of our study is that we show how implementing ITM performs in a real-world healthcare setting. In addition, our primary outcome is a patient-reported outcome measure widely considered to be the most appropriate marker for quality of postpartum recovery.^2,3^ Focusing on patient-reported outcome measures is important, as it bridges the gap between patient experience and clinical circumstances.^43^ With regard to baseline characteristics, there were more intrapartum caesarean deliveries in the ‘pre-ITM’ group and more scheduled caesarean deliveries in the ‘ITM’ group. Importantly, multivariate analysis showed that obstetric quality of recovery was better in the ITM group after adjusting for these confounders. The literature presents conflicting evidence on whether recovery outcomes are better or worse in repeat caesarean deliveries compared to primary caesarean deliveries.^44–46^ Recent work suggests that obstetric quality of recovery is similar in scheduled and nonscheduled caesarean deliveries, provided best practice recommendations are appropriately implemented.^47^ Consistent with these findings, we did not find a significant confounding effect of scheduled versus nonscheduled caesarean deliveries in multivariate analysis. Patients in the ‘pre-ITM’ group received short-acting intrathecal additives more frequently. This may reflect a hesitancy to administer three drugs for intrathecal administration, when this was not common practice previously. Importantly, despite the reduced use of adjuvants after ITM implementation, obstetric quality of recovery was significantly better, which emphasises the role of ITM in enhancing recovery outcomes. The most important limitation is that these data should not be interpreted as a randomised controlled trial. Even though our results were robust after performing a multivariate analysis correcting for potential confounders, results may have been influenced by differences in unmeasured covariates known to affect postoperative pain, such as ethnicity, socio-economic background as well as psychological characteristics.^48,49^ Furthermore, it is difficult to determine whether changes in opioid consumption and rescue analgesia requirements were due to ITM or changes in institutional factors not captured in this dataset, such as prescribing practices, nurse education surrounding pain management, and patient education and counselling, which could have had an impact of the use of these drugs. Also, the lower opioid consumption in the ITM group may be linked not only to better pain control with ITM but to reduced availability of rescue opioids, as the morphine tablets were nurse-administered. However, given the significant improvement in self-reported pain scores as measured with the ObsQoR-10, we find this to be unlikely. Finally, the pre-ITM group included patients managed with morphine PCA and epidural infusion which themselves, may have been associated with different analgesia outcomes compared with ITM.

As alternatives to ITM, other regional techniques such as transversus abdominis plane block, transversalis fascia plane block or local anaesthetic wound infiltration may have merits; however, these techniques fell outside the scope of this study.^50–52^

## Conclusion

In conclusion, implementing ITM in routine anaesthetic practice for patients undergoing caesarean delivery resulted in some improvements in quality of recovery, predominantly through improved pain control, physical comfort, independence and psychological wellbeing, despite an increase in pruritus. Multivariate analysis confirms these findings in the setting of intrapartum caesarean delivery. Other outcomes relevant to the recovery period, such as self-reported health score, the need for additional opioids as well as length of stay, also improved significantly. The reduction in length of stay of 4 h may hold clinical relevance, depending on hospital operational demands. Cautious interpretation is warranted in this nonrandomised implementation study. Future studies are needed to determine the impact on outpatient recovery metrics after hospital discharge.

## Supplementary Material

Supplemental Digital Content
