# Supplementary material for: The implementation of intrathecal morphine for caesarean delivery into clinical practice, and assessment of its impact on patient-reported quality of recovery using the ObsQoR-10-Dutch scale: A single-centre cohort study
Source: Eur J Anaesthesiol. 2025 Jan 29;42(4):332–9. doi: 10.1097/EJA.0000000000002127 (PMC11872268; doi:10.1097/EJA.0000000000002127)
Supplement: Supplemental Digital Content [file ejanet-42-332-s001.docx]

**Supplemental material 1: ObsQoR-10-Dutch questionnaire**

| **Hoe heeft u zich gevoeld in de afgelopen 24 uur?**  (op een schaal van 0 tot 10, omcirkel het juiste getal)   \| ***Hoeveel last heeft u  gehad van…*** \| 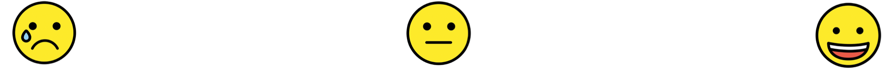  **Zeer ernstig** \| \| \| \| \| \| 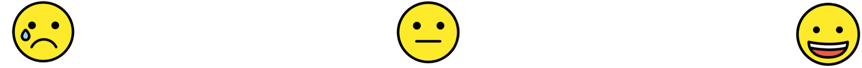  **Geen** \| \| \| \| \| \| \| --- \| --- \| --- \| --- \| --- \| --- \| --- \| --- \| --- \| --- \| --- \| --- \| --- \| \| 1. Pijn \| 10 \| 9 \| 8 \| 7 \| 6 \| 5 \| \| 4 \| 3 \| 2 \| 1 \| 0 \| \| 2. Misselijkheid of braken \| 10 \| 9 \| 8 \| 7 \| 6 \| 5 \| \| 4 \| 3 \| 2 \| 1 \| 0 \| \| 3. Duizeligheid \| 10 \| 9 \| 8 \| 7 \| 6 \| 5 \| \| 4 \| 3 \| 2 \| 1 \| 0 \| \| 4. Rillingen of trillen \| 10 \| 9 \| 8 \| 7 \| 6 \| 5 \| \| 4 \| 3 \| 2 \| 1 \| 0 \| \|  \|  \| \| \| \| \| \|  \| \| \| \| \| \| \|  \| **Helemaal niet mee eens** \| \| \| \| \| \| **Helemaal mee eens** \| \| \| \| \| \| \| 5. Ik heb me comfortabel gevoeld. \| 0 \| 1 \| 2 \| 3 \| 4 \| 5 \| \| 6 \| 7 \| 8 \| 9 \| 10 \| \| 6. Ik kan zelfstandig mobiliseren en/of uit bed komen. \| 0 \| 1 \| 2 \| 3 \| 4 \| 5 \| \| 6 \| 7 \| 8 \| 9 \| 10 \| \| 7. Ik kan mijn baby vasthouden zonder hulp. \| 0 \| 1 \| 2 \| 3 \| 4 \| 5 \| \| 6 \| 7 \| 8 \| 9 \| 10 \| \| 8. Ik kan mijn baby voeden/verzorgen zonder hulp. \| 0 \| 1 \| 2 \| 3 \| 4 \| 5 \| \| 6 \| 7 \| 8 \| 9 \| 10 \| \| 9. Ik kan mezelf verzorgen (dagelijkse hygiëne en toiletgang). \| 0 \| 1 \| 2 \| 3 \| 4 \| 5 \| \| 6 \| 7 \| 8 \| 9 \| 10 \| \| 10. Ik heb de controle. \| 0 \| 1 \| 2 \| 3 \| 4 \| 5 \| \| 6 \| 7 \| 8 \| 9 \| 10 \| |
| --- | --- | --- | --- | --- | --- | --- | --- | --- | --- | --- | --- | --- | --- | --- | --- | --- | --- | --- | --- | --- | --- | --- | --- | --- | --- | --- | --- | --- | --- | --- | --- | --- | --- | --- | --- | --- | --- | --- | --- | --- | --- | --- | --- | --- | --- | --- | --- | --- | --- | --- | --- | --- | --- | --- | --- | --- | --- | --- | --- | --- | --- | --- | --- | --- | --- | --- | --- | --- | --- | --- | --- | --- | --- | --- | --- | --- | --- | --- | --- | --- | --- | --- | --- | --- | --- | --- | --- | --- | --- | --- | --- | --- | --- | --- | --- | --- | --- | --- | --- | --- | --- | --- | --- | --- | --- | --- | --- | --- | --- | --- | --- | --- | --- | --- | --- | --- | --- | --- | --- | --- | --- | --- | --- | --- | --- | --- | --- | --- | --- | --- | --- | --- | --- | --- | --- | --- | --- | --- | --- | --- | --- | --- | --- | --- | --- | --- | --- | --- | --- | --- | --- | --- | --- | --- | --- | --- | --- | --- | --- | --- | --- | --- | --- | --- | --- | --- | --- | --- | --- |

**Supplemental material 2: Validation of ObsQoR-10-Dutch**

*Translation from ObsQoR-10-English into ObsQoR-10-Dutch*

The English ObsQoR-10 questionnaire^1^ was translated into Dutch for the population of the Netherlands, in accordance with the methodology for cross-cultural adaptation of self-reported measures.^2^ First, this involved the translation of the ObsQoR-10 into Dutch by two authors fluent in Dutch and English (OvdB, MR). Second, the translated Dutch ObsQoR-10 was translated back into English by a native English speaker fluent in Dutch not affiliated with this study (KdRB). Third, the backtranslation was compared to the original questionnaire (KdRB) and any disagreements were resolved through consensus. Lastly, the Dutch ObsQoR-10 questionnaire was discussed face-to-face with five healthcare professionals and five pregnant patients to collect feedback that could lead to further modifications. This feedback did not lead to additional modifications and the translated Dutch ObsQoR-10 is provided as Supplementary Material 1.

*Validation of ObsQoR-10-Dutch: methods*

The validation of ObsQoR-10-Dutch involved a comprehensive psychometric evaluation in accordance with the appropriate guideline, assessing both validity and reliability.^3^ Validity was assessed by evaluating (i) convergent validity (comparing ObsQoR-10-Dutch to GHVAS score), (ii) discriminant validity (comparing ObsQoR-10-Dutch between women reporting good recovery (GHVAS ≥ 70) vs poor recovery (GHVAS < 70)) and (iii) hypothesis testing (association between ObsQoR-10-Dutch and ASA physical status, maternal age, body mass index, blood loss, length of hospital stay and urgency of caesarean delivery). Reliability was assessed by evaluating (i) internal consistency (Cronbach alpha and inter-item correlation for the 10 individual items), (ii) split-half reliability (random split segments), (iii) test-retest reliability (in 10 patients invited to repeat the questionnaire after 60 min) and (iv) floor and ceiling effects (<15% respondents achieving the highest or lowest possible score).

*Validation of ObsQoR-10-Dutch: validity*

With regard to convergent validity, the ObsQoR-10-Dutch scores correlated moderately with general health scores with coefficient 0.565 (95% CI: 0.417 to 0.684; *P* < 0.001). With regard to discriminant validity, ObsQoR-10-Dutch scores were significantly higher in those with good recovery (GHVAS ≥ 70) than in those with poor recovery (GHVAS < 70); 77 ± 11 vs 64 ± 15 (*P* < 0.001). With regard to construct validity using hypothesis testing, ObsQoR-10-Dutch scores correlated inversely with ASA physical status (r=-0.204; 95 CI, -0.380 to -0.013; *P* = 0.036), correlated with maternal age (r=0.210; 95% CI, 0.020 to 0.386; *P*=0.030) and correlated inversely with length of hospital stay (r=-0.264; 95% CI, -0.433 to -0.077; *P*=0.006) but not with body mass index (r=0.114; 95% CI, -0.079 to 0.298; *P*=0.245) or blood loss (r=-0.102; 95% CI, -0.287 to 0.090; *P*=0.297). ObsQoR-10 scores were significantly higher in patients undergoing scheduled caesarean delivery compared to participants undergoing non-scheduled caesarean delivery (73 ± 12 vs 64 ± 17, *P*=0.001).

*Validation of ObsQoR-10-Dutch: reliability*

With regard to internal consistency of the 10 items on the ObsQoR-10-Dutch questionnaire, the Cronbach alpha was 0.769, which is considered acceptable, and the inter-item correlation for the individual items is shown in a heatmap (see below). With regard to split-half reliability, the Spearman-Brown coefficient was 0.810, which is considered good. With regard to test-retest reliability, the agreement interclass coefficient was 0.960, which is considered excellent. With regard to floor and ceiling effects, no participants scored 0/100 and one participant (1.0%) scored 100/100.

*Heat map of inter-item correlations*

|  | **Q1** | **Q2** | **Q3** | **Q4** | **Q5** | **Q6** | **Q7** | **Q8** | **Q9** | **Q10** |
| --- | --- | --- | --- | --- | --- | --- | --- | --- | --- | --- |
| **Q1 pain** | 1 | 0.215 | 0.241 | 0.174 | 0.328 | 0.393 | 0.197 | 0.170 | 0.220 | 0.246 |
| **Q2 nausea** | 0.215 | 1 | 0.375 | 0.350 | 0.078 | 0.036 | 0.258 | 0.193 | 0.050 | 0.182 |
| **Q3 dizziness** | 0.241 | 0.375 | 1 | 0.341 | 0.091 | 0.036 | 0.048 | 0.021 | -0.008 | 0.182 |
| **Q4 shivering** | 0.174 | 0.350 | 0.341 | 1 | 0.133 | 0.114 | 0.201 | 0.037 | 0.160 | 0.037 |
| **Q5 comfortable** | 0.328 | 0.078 | 0.091 | 0.133 | 1 | 0.331 | 0.171 | 0.230 | 0.326 | 0.275 |
| **Q6 mobilise** | 0.393 | 0.036 | 0.036 | 0.114 | 0.331 | 1 | 0.412 | 0.397 | 0.734 | 0.453 |
| **Q7 holding baby** | 0.197 | 0.258 | 0.048 | 0.201 | 0.171 | 0.412 | 1 | 0.665 | 0.457 | 0.432 |
| **Q8 feeding baby** | 0.170 | 0.193 | 0.021 | 0.037 | 0.230 | 0.397 | 0.665 | 1 | 0.506 | 0.481 |
| **Q9 hygiene** | 0.220 | 0.050 | -0.008 | 0.160 | 0.326 | 0.734 | 0.457 | 0.506 | 1 | 0.562 |
| **Q10 control** | 0.246 | 0.182 | 0.182 | 0.037 | 0.275 | 0.453 | 0.432 | 0.481 | 0.562 | 1 |

Heatmap of inter-item correlations for the ObsQoR-10-Dutch score at 24 h after caesarean delivery. The 10 questions composing the ObsQoR-10-Dutch questionnaire (from Q1 to Q10) are distributed on the axes. The strength of the correlation between the items is represented by the colour shade (from red for very strong association, to green for very weak association).

**REFERENCES**

1. Sultan P, Kormendy F, Nishimura S, *et al.* Comparison of spontaneous versus operative vaginal delivery using Obstetric Quality of Recovery-10 (ObsQoR-10): An observational cohort study. *Journal of Clinical Anesthesia* 2020; **63**:109781.

2. Beaton DE, Bombardier C, Guillemin F, *et al.* Guidelines for the Process of Cross-Cultural Adaptation of Self-Report Measures. *Spine* 2000; **25**:3186–3191.

3. Mokkink LB, Terwee CB, Patrick DL, *et al.* The COSMIN checklist for assessing the methodological quality of studies on measurement properties of health status measurement instruments: an international Delphi study. *Quality of Life Research* 2010; **19**:539–549.

**Supplemental material 3: Postpartum recovery outcomes following caesarean delivery under spinal anaesthesia with and without neonatal admission to the neonatal intensive care unit**

|  | **No NICU**  **n=102** | **NICU**  **n=40** | ***P* value** |
| --- | --- | --- | --- |
| ObsQoR-10 at 24 h (0-100) ^a^ | 69 ± 15 | 66 ± 19 | 0.351 |
| 1 Pain | 5 [3 to 7] | 5 [3 to 6] | 0.217 |
| 2 Nausea | 1 [0 to 4] | 0 [0 to 2] | 0.152 |
| 3 Dizziness | 2 [0 to 5] | 0 [0 to 4] | 0.068 |
| 4 Shivering | 1 [0 to 4] | 2 [0 to 4] | 0.386 |
| 5 Comfort | 8 [6 to 9] | 8 [5 to 9] | 0.843 |
| 6 Mobilization | 8 [5 to 9] | 7 [6 to 9] | 0.771 |
| 7 Hold baby | 9 [6 to 10] | 7 [3 to 10] | 0.023 |
| 8 Feed baby | 7 [4 to 8] | 4 [0 to 7] | 0.004 |
| 9 Hygiene | 7 [5 to 9] | 7 [4 to 9] | 0.933 |
| 10 Control | 8 [5 to 8] | 7 [5 to 9] | 0.830 |
| GH-VAS at 24 h (0-100) | 62 ± 18 | 59 ± 20 | 0.248 |
| Length of stay (hours) ^b^ | 40 [35 to 51] | 52 [45 to 56] | < 0.001 |
| Oral morphine equivalents in 24 h (mg) | 0 (0 to 36, 0 to 171) | 9 (0 to 27, 0 to 162) | 0.535 |

Data are shown as median [IQR], median [IQR, range] or mean ± SD. ^a^ sum of the 10 questions, scores of questions 1-4 are inverted, ^b^ from start of caesarean delivery until maternal hospital discharge, ^c^ median [IQR, range]. GH-VAS, general health measured by visual analogue score.
